# Effect of Lysyl Oxidase G473A Polymorphism on Lysyl Oxidase and Total Soluble Collagen Expression in Oral Submucous Fibrosis

**DOI:** 10.31557/APJCP.2021.22.8.2493

**Published:** 2021-08

**Authors:** Sanjit Mukherjee, Atul Katarkar, Richa Dhariwal, Sweta Mohanty, Basudev Mahato, Jay Gopal Ray, Keya Chaudhuri

**Affiliations:** 1 *CSIR-Indian Institute of Chemical Biology, Kolkata, India. *; 2 *Department of Oral Pathology, Dr. R. Ahmed Dental College and Hospital, Kolkata, India. *

**Keywords:** Oral submucous fibrosis, lysyl oxidase, collagen, single nucleotide polymorphism

## Abstract

**Background:**

Oral submucous fibrosis (OSF) is a debilitating collagen-metabolic disorder leading to submucosal fibrosis and trismus. Lysyl oxidase (LOX), a critical collagen biosynthetic enzyme, is up-regulated in OSF. Polymorphisms in the Lysyl oxidase gene have been associated with increased risk of OSF and might affect normal collagen synthesis, accumulation, or degradation, crucial in determining fibrosis severity.

**Methods:**

One hundred OSF cases and 100 controls were genotyped for LOX G473A(Arg158Gln) polymorphism by polymerase chain reaction-restriction fragment length polymorphism. The expression of LOX was estimated both by quantitative mRNA analysis and western blot. Total soluble collagen was evaluated from mucosal tissue obtained from OSF cases. Immunohistochemical (IHC) localization of type 1 collagen was performed in mucosal tissue obtained from patients carrying various genotypes.

**Results:**

Heterozygous G473A genotype was significantly higher in OSF cases [2.063(95% CI =1.059-4.016)], among 26-40 years age-group [4.375(95% CI=1.323-14.267),p=0.029] and in male patients [2.38 (95% CI= 1.107-5.121), p= 0.042]. LOX expression was significantly higher in cases of the heterozygous or homozygous carrier (p<0.001). We found the total soluble collagen level significantly (p<0.001) higher among patients carrying GA or AA genotype. IHC revealed focal deposition of type1 collagen in the submucosal tissue; comparatively higher deposition was evident in mucosal tissue of OSF patients carrying AA genotype.

**Conclusions:**

These findings suggest LOX G473A polymorphism confers an increased risk of OSF and may affect collagen accumulation in OSF cases.

## Introduction

Oral submucous fibrosis (OSF) is a chronic, insidious, progressive, scarring disease of the oral mucosa that affects the entire oral cavity, sometimes extending to the faucial pillars pharynx. It is characterized by a fibrotic change in the submucosal layer with progressive tissue rigidity leading to restricted mouth opening, causing ‘trismus.’ This oral potentially malignant disorder has a high rate of malignant transformation (7%-30%) to oral squamous cell carcinoma (Arora et al., 2014) (More et al., 2020). The etiology of OSF is elusive. Arecoline, an alkaloid of areca nut released during chewing, is mainly regarded as the causal factor of OSF. Arecoline is the most potent agent which induces an abnormal increase in collagen production (Xia et al., 2009). Lysyl Oxidase (LOX) is a copper-dependent amine oxidase and critical collagen biosynthetic enzyme (Rucker et al., 1998). Arecanut contains a high amount of soluble copper and may attribute to the upregulation of LOX, leading to increased collagen synthesis in OSF (Kuo et al., 1995; Trivedy et al., 1999; Peng et al., 2020). LOX is produced initially as a pre-proenzyme and then converted to proenzyme LOX (pLOX) that finally cleaves between Glycine-168 and Aspartic acid-169 by peptidase to yield an active/mature LOX (aLOX) (Smith et al., 2016). A single nucleotide polymorphism of G473A of *LOX* gene (rs1800449) changes Arginine at residue 158 to Glutamine (LOX Arg158Gln), a highly conserved region near the peptidase cutting sites of LOX. The minor A-allele is associated with an increased risk of OSF (Shieh et al., 2007; Shieh et al., 2009), cancers, and other diseases (Wang et al., 2012; Friesenhengst et al., 2014; Gao et al., 2015). Very few studies exist documenting the presence of LOX G473A polymorphism in Indian populations. Hence, the present study examines the association of LOX G473A polymorphism with OSF in a population from eastern India and the expression of LOX and type 1 collagen among different genotypes. 

## Materials and Methods

*Study population*

One hundred consecutive cases (male = 75, females = 25), newly diagnosed with OSF, confirmed histopathologically at Dr. R Ahmed Dental College and Hospital, Kolkata, India, between November 2011- January 2014, were selected for this observational study. The Control group included 100 (male=72, female=28) (Table 1) volunteers without any mucosal lesions or other systemic conditions, and a habit of chewing betel quid with or without tobacco for more than one year. The inter-incisal distance or mouth opening of OSF cases was recorded and was graded as mild (>35mm), moderate ( 20- 35 mm), and severe (< 20mm) cases (Kiran Kumar et al., 2007). A blood sample of 2-5 ml was collected from the antecubital vein from all the cases and controls. Written consent was obtained from all subjects before collecting blood or tissue, and the study was duly approved by the institutional ethical committee as per the guidelines. 

*DNA isolation and genotyping analysis of LOX G473A*

**Figure 1:**
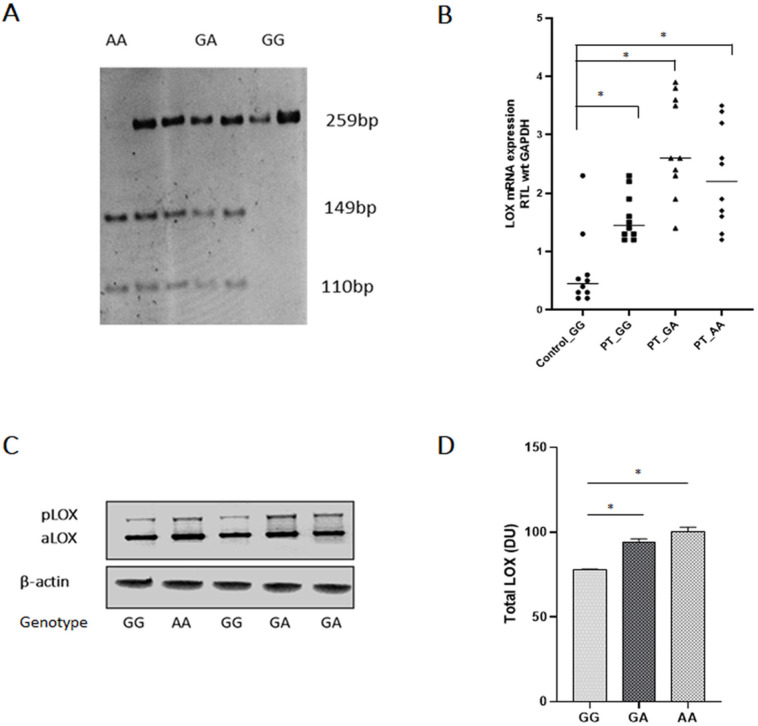
A, Representative PCR-RFLP image for determining LOX G473A genotype; B, qRT-PCR of LOX mRNA expression in normal wild-type and OSF cases. Significantly higher LOX expression was found in patients carrying GA or AA genotype (mean±SEM); C, Representative Western blots of LOX enzyme expression in oral mucosa of cases; D, Densitometric analysis of total LOX expression in oral mucosa of OSF cases (mean±SEM). (* p<0.001); GG-wild type; GA-heterozygous; AA-homozygous; PT- patient; bp- base pair, KD- kilodalton

Genomic DNA was isolated from the peripheral blood by proteinase-K treatment and salt extraction method (Sambrook, 2001). Genotyping of the *LOX *gene encompassing the polymorphic region (G473A) was done using polymerase chain reaction (PCR) using primers forward: 5`-CACTGGTTCCAAGCTGGCTA-3` reverse:5`-GGAAGTAGCCAGTGCCGTAT-3`. The amplified PCR product was then subjected to restriction fragment length polymorphism (RFLP) detection by subsequent digestion with restriction enzyme Pst1(CTGCAG) and was then resolved on 6% polyacrylamide gel. Wild (GG), homozygous mutant (AA), and heterozygous mutant (GA) gave one (259 bp), two (149 and 110 bp), and three (259, 149, 110 bp) bands, respectively (Figure 1A). 

*RNA extraction and quantitative RT-PCR*

Oral biopsy tissues were collected from the lesion of OSF cases. Normal tissue was collected from the gingiva of healthy controls undergoing 3rd molar extraction. The tissues were washed in ice-cold 1X Phosphate buffer saline in 1.5ml microcentrifuge. According to the manufacturer’s protocol, total RNA was extracted from a part of the biopsied tissue using TRIZOL reagent (Invitrogen, USA). We performed quantitative real-time reverse transcriptase-polymerase chain reaction (qRT-PCR) using One-Step SYBR® Ex Taq ™ qRT-PCR Kit (Takara, Japan). We amplified the polymorphic region using the following primers LOX forward 5’-GATCCTGCTGATCCGCGACAA-3’; reverse 5’-GGGAGACCGTACTGGAAGTAG-3’. GAPDH was used as an internal control using the following primers, forward 5’-ATGGGGAAGGTGAAGGTCGG-3’; reverse 5’-GGATGCTAAGCAGTTGGT-3’. Thermal cycling was performed in a BioRad iQ5 Real-Time PCR Detection System (BioRad, USA).

*Protein extraction and Western Blot analysis*

Total protein from a part of the biopsied tissue was extracted using radioimmunoprecipitation assay lysis buffer (1X) containing protease inhibitor cocktail (Roche, USA), and concentration was estimated by Bradford dye-binding method using the Bio-Rad protein assay kit (BioRad Inc, USA). 50µg protein from each sample was separated on 12% Sodium dodecyl sulfate-polyacrylamide gel electrophoresis. Western blotting was done using rabbit polyclonal anti-LOX (1:1000, Novus Biologicals, USA), mouse β-actin antibodies (1:2000, Sigma-Aldrich) and mouse monoclonal COL1A Antibody (COL-1) (Santacruz Biotechnology, Dallas, USA); followed by incubation with alkaline phosphatase-conjugated goat anti-rabbit *IgG* (GENEI) or rabbit anti-mouse *IgG* (GENEI). The alkaline phosphatase-positive bands were visualized using a 5-bromo,4-chloro,3-indolylphosphate/nitrobluetetrazolium (*BCIP/NBT*) (GENEI). The band’s intensities were analyzed using ImageJ (http://rsbweb.nih.gov/ij/download.html) and expressed in densitometric units (DU).

*Total collagen assay*

Total collagen from the biopsied tissue portions was assayed using the Soluble collagen assay Sircol kit (Biocolor, Northern Ireland) following the manufacturer’s protocol. 50µg of protein homogenate was used for this assay. 

*Immunohistochemistry*

Immunostaining to detect type 1 collagen was performed using Novolink polymer detection system (Leica Microsystems, Switzerland) following manufacturer protocol. Briefly, oral biopsy specimens were paraffin-embedded, and 4-5 µm thickness sections were collected on poly-L-lysine coated slides. The slides were rehydrated and treated with citrate buffer for antigen retrieval. Endogenous peroxide activity was neutralized using the peroxidase block. The sections were then incubated with an anti-collagen I antibody (Abcam, USA) and developed with Novolink polymer provided in the kit. DAB substrate buffer was used to develop peroxidase activity and visualize. The sections were then counterstained with hematoxylin, and multiple fields were observed under LEICA DM 3000 microscope (Leica Microsystems, Switzerland) for evaluation. 

*Statistical Analysis*

The odds ratio (OR) and 95% CI (confidence interval) were calculated using JAVASTAT 2-way contingency table analysis software (http://statpages.org/ctab2x2.html). The Student’s t-test was performed to observe the differences between protein expressions, and the p-value was calculated. A p-value of <0.05 was considered significant.

## Results

*Demographic variables and association of LOX G473A polymorphism with OSF*

The demographic variables are summarized in Table 1. Out of 100 OSF patients (cases) recruited for the present study, 75% were male, and 25% were female. The study population was categorized according to age as young (<25 years), middle-aged (26-40 years), and older (> 40 years) age groups. The majority of male patients were <25 years of age (52%), and females belong to 26-40 years(50%). Further, most cases (51%) were in clinical-grade II, followed by 43% in grade III and 6% in grade I and (Table 1). About 58.8% of males had areca nut chewing habits with/without tobacco, 41.4 % had combined chewing habits. Incidentally, all the females OSF patients were areca nut chewers. Overall the frequency of minor A allele in our study population was 17.5% (70 out of 400) and was a significantly higher prevalence in OSF cases compared to controls [OR=1.888, 95% CI(1.110-3.209); p=0.018]. A higher frequency of heterozygous GG (30%) and homozygous mutant AA (7%) genotype was found in OSF cases compared to the controls (18%, 4%). The increase in heterozygous frequency was statistically significant [OR=2.063, 95% CI(1.059-4.016); (p=0.049)] (Table 2). Age stratified analysis indicated a statistically significant [OR=4.375, 95% CI (1.323-14.267); p=0.029] increase in heterozygous genotype frequency in the 26-40 years age group. In <25 years age group, marginal increase in heterozygous genotype was observed [OR=2.75, 95% CI (0.823-9.042); p=0.178) (Table 2). Moreover, a statistically significant higher frequency of heterozygous genotype was seen in male cases [OR =2.38, 95% CI (1.107-5.121); p= 0.042]. No significant association was detected between genotype and the chewing habit (areca nut/combined habit) (Table 2). Though not statistically significant, the wild-type GG genotype percentage was higher in grade III cases (67.4%) than in grade II (56.8%). In comparison, the homozygous mutant genotype in grade II (7.9%) was higher compared to grade III (4.7%) (Table 2). Moreover, the prevalence of wild-type genotype was 58%, 63%, and 93% respectively among patients with exposure of <5, 6-10 and >10 years of chewing habit, whereas heterozygous genotype was found in 34% and 37% patients with <5 and 6-10 years of chewing habit respectively (Table 2).

*LOX expression in OSF patients*

Mucosal tissue was collected from ten patients from each group carrying wild-types, heterozygous, and homozygous mutant genotypes from the same lesion site. Ten gingival tissue was obtained from control individuals carrying wild-type genotype during their 3^rd^ molar extraction. LOX mRNA expression in mucosal tissues from the site of the lesion of cases was significantly higher than healthy tissue (Figure 1B). Western blot revealed significantly higher total LOX protein levels in OSF cases carrying heterozygous or homozygous genotype than wild-type cases (Figure 1C and D). 

*Collagen expression in patients carrying LOX G473A polymorphism*

**Figure 2:**
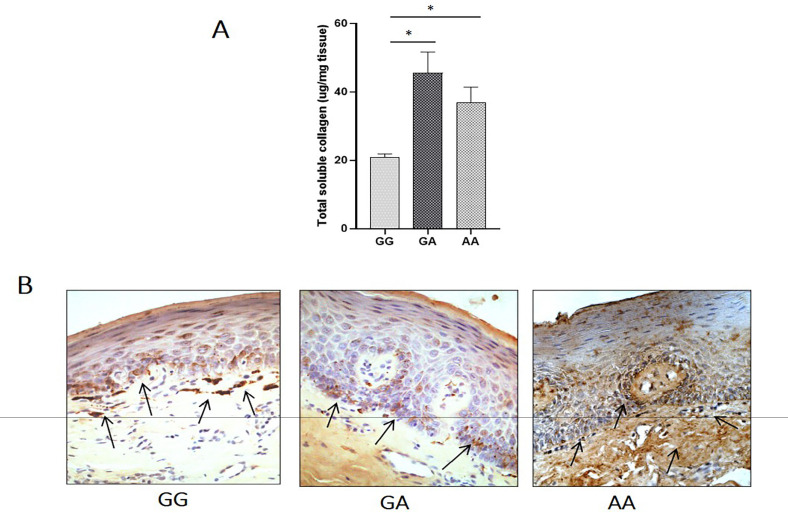
A, Total collagen content in the oral mucosal tissues collected from OSF cases. (mean±SEM) (* p<0.001) B, Immunohistochemical localization of type 1 collagen in oral mucosal tissues. Arrows show the deposition of collagen

We observed a 2-fold and 1.5 fold higher accumulation of total soluble collagen in OSF tissues carrying heterozygous (45.54±2.75 µg/mg tissue) (mean±SEM) or homozygous (36.9±2.028 µg/mg tissue) mutant genotype, compared to the OSF tissues carrying wild-type genotype (20.9±1.01 µg/mg tissue). Type-1 collagen was immunohistochemically localized in OSF tissues carrying wild-type, heterozygous or homozygous genotype. OSF tissues carrying a wild-type genotype revealed focal droplets of collagen accumulation at the submucosal level (as marked by arrows) (Figure 2 B). Diffused and higher deposition of type 1 collagen was observed in heterozygous tissues, which was even higher in homozygous tissues than wild-type tissues.

**Table 1:** Demographic Status of the Cases and Controls of the Study Population

Parameters	Males N(%)		Females N(%)		Total N(%)	
	Cases	Controls	Cases	Controls	Cases	Controls
	(N=75)	(N=72)	(N=25)	(N=28)	(N=100)	(N=100)
Age (Years)						
0-25	39 (52.0)	21 (29.1)	5 (20)	5 (17.8)	44	26
26-40	32 (42.6)	23 (31.9)	10 (40)	14 (50)	42	37
>40	4.0 (5.30)	28 (38.8)	10 (40)	9 (32.14)	14	37
OSF grade (IID)	(N=75)		(N=25)		(N=100)	
I-Mild (>35mm)	3 (4)		3 (12)		6 (6)	
II-Moderate (20-35mm)	41(54.6)		10 (40)		51 (51)	
III-Severe (<20mm)	31(41.3)		12 (48)		43(43)	
Habit	(N=75)		(N=25)		(N=100)	
Areca Nut	44 (58.6)	72	25 (100)		69 (69)	100
Combined habit	31(41.4)		0		31(31)	

N, Sample size; IID, INteriNcisal distance

**Table 2:** Distribution of Lysyl Oxidase G473A Genotype among OSF Patients and Controls

LOX genotype distribution in the total study sample				
Genotype	Cases N (%)	Control N (%)	OR (95 CI)	P value
Wild (GG)	63 (63)	78 (78)	Reference	
Heterozygous (GA)	30 (30)	18 (18)	2.063 (1.059-4.016)	0.049*
Mutant (AA)	7 (7)	4 (4)	2.167 (0.645-7.245)	0.368
Allele frequency in the total study sample				
G	156 (78)	174 (87)	Reference	
A	44 (22)	26 (13)	1.888 (1.110-3.209)	0.018*
LOX genotype distribution according to age groups				
<25 Years	Cases (n=44)	Control (n=26)	OR (95 CI)	P value
C	28 (63.6)	22 (84.6)	Reference	
GA	14 (31.8)	4 (15.4)	2.75 (0.823-9.042)	0.178
AA	2 (4.6)	0	Inf (0.382-Inf)	0.613
26-40 Years	Cases (N=42)	Control (N=37)	OR (95 CI)	P value
GG	24 (57.1)	30 (81.1)	Reference	
GA	14 (33.3)	4 (10.8)	4.375 (1.323-14.267)	0.029*
AA	4 (9.6)	3 (8.1)	1.667 (0.376-7.337)	0.817
>40 Years	Cases (N=14)	Control (N=37)	OR (95 CI)	P value
GG	11 (78.6)	26 (70.3)	Reference	
GA	2 (14.3)	10 (27.0)	0.473 (0.101-2.295)	0.607
AA	1 (7.1)	1 (2.7)	2.364 (0.227-24.723)	1
LOX genotype distribution according to gender				
Males	Cases (n=75)	Control (n=72)	OR (95 CI)	P value
GG	46 (61.3)	57 (79.2)	Reference	
GA	25 (33.3)	13 (18.1)	2.38 (1.107-5.121)	0.042*
AA	4 (5.4)	2 (2.7)	2.478 (0.503-12.065)	0.529
Females	Cases (n=25)	Control (n=28)	OR (95 CI)	P value
GG	17 (68)	20 (71.4)	Reference	
GA	5 (20)	6 (21.4)	0.98 (0.267-3.62)	1
AA	3 (12)	2 (7.2)	1.765 (0.309-9.898)	0.91
Habit-wise genotype distribution				
Genotype	Areca nut (n=69)	Combined habit (n=31)	OR (95 CI)	P value
GG	42 (60.9)	21 (67.7)	Reference	
GA	22 (31.9)	8 (25.8)	1.375 (0.532-3.532	0.682
AA	5 (7.2)	2 (6.5)	1.250 (0.253-6.023)	1
Distribution of genotype according to grade				
Genotype	Grade I (N=6)	Grade II (N=51)	Grade III (N=43)	
GG	5 (83.3)	29 (56.8)	29 (67.4)	
GA	Nil	18 (35.3)	12 (27.9)	
AA	1 (16.6)	4 (7.9)	2 (4.7)	
Distribution of genotypes according to exposure to habit				
Exposure (yrs)	Total (n=100)	GG	GA	AA
0-5 years	59	33 (58)	20 (34)	6 (10)
6-10 years	27	17 (63)	10 (37)	Nil
>10 years	14	13 (93)	Nil	1 (7)

N(%) - Sample size (%)

## Discussion

In this study, both the OSF cases and controls had a history of chewing areca nut with or without smoking and chewing tobacco. Primarily the study population belonged to an urban origin and similar socio-economic background. Previously cohort-based studies reported that the presence of LOX G473A (Arg158Gln) polymorphism increased OSF susceptibility in some populations. Hence, firstly, we were interested in examining this genotype’s predisposition among OSF cases in an eastern Indian population. The second interesting aspect of our study was investigating whether the presence of LOX G473A (Arg158Gln) predisposition affects the patients’ LOX and collagen accumulation.

This study observed that the minor A allele frequency was 17.5% in our total study population. This observation was contrary to a previously published research (Thorawat et al., 2014) that did not find any minor A allele in an Indian population. It should be noted that the study was carried out in a smaller cohort selected by ‘convenience sampling’. Only a few studies have explored the role of LOX G473A polymorphism in various diseases in the Indian population (Pichu et al., 2017) (Sathyan et al., 2013) (Ray et al., 2013) with much larger cohort sizes. Our observation was similar to the previously reported LOX G473A polymorphism frequency observed in these studies. We found the minor A allele to be significantly higher in the cases. Heterozygous LOX genotype was significantly higher in the middle-age group (26-40 years) and male OSF cases. This observation goes in line with Shieh et al. among 83 OSF patients (Shieh et al., 2009). This finding can be substantiated based on data indicating a higher prevalence of chewing habit and thus OSF among men than in women (Hazarey et al., 2007) (Ranganathan et al., 2004). Overall, LOX G473A (Arg158Gln) confers significant risk in developing OSF.

Our next goal was to find the effect of LOX G473A (Arg158Gln) polymorphism upon LOX expression. We found LOX levels were significantly higher in cases carrying GA or AA genotype compared to wild-type cases. The Arg158Gln site is near the procollagen peptidase cutting site of pLOX, just ten amino acid positions away between Gln168 and Arp169, and may affect the peptidase cutting site of LOX. A further study regarding the protein sequence can provide a better explanation.

Our findings support the hypothesis that the accumulation of collagen seen in advanced OSF cases is not only due to fibrogenesis but may also be due to a decrease in the breakdown of collagen earlier cross-linked by LOX and thus overall turnover (Rajalalitha and Vali, 2005). This cross-linking of collagen fibrils may be affected by various polymorphic sites of LOX (Consuegra and Johnston, 2006). However, its exact role is still elusive. To conclude, additional studies are required to uncover the mechanistic role of LOX and its genetic polymorphisms concerning the severity of fibrosis and pathogenesis.

## Author Contribution Statement

SM and KC contributed to design, methodology, data collection, analysis, manuscript writing and critical review; AK contributed to methodology data collection and analysis; SwM, RD and BM contributed to data collection; JGR contributed to review of manuscript.
